# Insulin Sensitivity Is Retained in Mice with Endothelial Loss of Carcinoembryonic Antigen Cell Adhesion Molecule 1

**DOI:** 10.3390/cells10082093

**Published:** 2021-08-14

**Authors:** Harrison T. Muturi, Saja S. Khuder, Hilda E. Ghadieh, Emily L. Esakov, Hyelim Noh, Heejoon Kang, Marcia F. McInerney, Jason K. Kim, Abraham D. Lee, Sonia M. Najjar

**Affiliations:** 1 Department of Biomedical Sciences, Heritage College of Osteopathic Medicine, Ohio University, Athens, OH 45701, USA; muturi@ohio.edu (H.T.M.); hg36@aub.edu.lb (H.E.G.); 2 Center for Diabetes and Endocrine Research, College of Medicine and Life Sciences, University of Toledo, Toledo, OH 43606, USA; saja.khuder@gmail.com (S.S.K.); esakove@ccf.org (E.L.E.); marcia.mcinerney@utoledo.edu (M.F.M.); 3 Department of Medicinal and Biological Chemistry, College of Pharmacy and Pharmaceutical Sciences, Toledo, OH 43606, USA; 4 Program in Molecular Medicine, University of Massachusetts Medical School, Worcester, MA 01605, USA; HyeLim.Noh@crl.com (H.N.); sgndoc@nate.com (H.K.); Jason.Kim@umassmed.edu (J.K.K.); 5 Department of Breast-Endocrine Surgery, Dongtan Sacred Heart Hospital, Hallym University College of Medicine, Hwaseong 18450, Korea; 6 Division of Endocrinology, Metabolism and Diabetes, University of Massachusetts Medical School, Worcester, MA 01605, USA; 7 Department of Rehabilitation Sciences, Judith Herb College of Education, Human Science and Human Service, The University of Toledo, Toledo, OH 43606, USA; Abraham.lee2@utoledo.edu; 8 Diabetes Institute, Heritage College of Osteopathic Medicine, Ohio University, Athens, OH 45701, USA

**Keywords:** carcinoembryonic antigen-related cell adhesion molecule-1, insulin transport, insulin clearance, insulin resistance, normo-insulinemia

## Abstract

CEACAM1 regulates endothelial barrier integrity. Because insulin signaling in extrahepatic target tissues is regulated by insulin transport through the endothelium, we aimed at investigating the metabolic role of endothelial CEACAM1. To this end, we generated endothelial cell-specific *Ceacam1* null mice (*VECadCre+Cc1^fl/fl^*) and carried out their metabolic phenotyping and mechanistic analysis by comparison to littermate controls. Hyperinsulinemic-euglycemic clamp analysis showed intact insulin sensitivity in *VECadCre+Cc1^fl/fl^* mice. This was associated with the absence of visceral obesity and lipolysis and normal levels of circulating non-esterified fatty acids, leptin, and adiponectin. Whereas the loss of endothelial *Ceacam1* did not affect insulin-stimulated receptor phosphorylation, it reduced IRS-1/Akt/eNOS activation to lower nitric oxide production resulting from limited SHP2 sequestration. It also reduced Shc sequestration to activate NF-κB and increase the transcription of matrix metalloproteases, ultimately inducing plasma IL-6 and TNFα levels. Loss of endothelial *Ceacam1* also induced the expression of the anti-inflammatory CEACAM1-4L variant in M2 macrophages in white adipose tissue. Together, this could cause endothelial barrier dysfunction and facilitate insulin transport, sustaining normal glucose homeostasis and retaining fat accumulation in adipocytes. The data assign a significant role for endothelial cell CEACAM1 in maintaining insulin sensitivity in peripheral extrahepatic target tissues.

## 1. Introduction

Carcinoembryonic antigen-related cell adhesion molecule-1 (CEACAM1) is a transmembrane glycoprotein that undergoes phosphorylation by insulin and vascular endothelial growth factor receptor-2 (VEGFR2) [1,2]. It is highly expressed in hepatocytes, where it promotes insulin clearance to sustain insulin sensitivity [3]. This is mediated by partaking in the insulin receptor internalization complex and increasing the rate of its cellular uptake. Consistently, mice with global and liver-specific null deletion of *Ceacam1* gene [4,5] and with its liver-specific inactivation [6] exhibited hyperinsulinemia with resultant insulin resistance, elevated hepatic lipid production, and redistribution to white adipose tissue to contribute to visceral adiposity. *Cc1*^−/−^ mice also manifested dysregulated endothelial and cardiovascular functions [7], which, together with metabolic abnormalities [8], were reversed with exclusive reconstitution of CEACAM1 in hepatocytes [7].

The endothelial cells of the liver capillaries are fenestrated to allow rapid insulin transport into hepatocytes. In contrast, the endothelial layer lining the vasculature of skeletal muscle and adipose tissue is continuous and forms an endothelial barrier that regulates insulin transport to the interstitial space to ultimately bind to its receptors on myocytes [9] and adipocytes [10]. Insulin transport occurs either transcellularly via receptor-mediated insulin transcytosis or paracellularly via tight junctions between neighboring endothelial cells [9]. Transcytosis in endothelial cells supports the notion that insulin does not undergo significant degradation in endothelial cells [11]. Of note, receptor-mediated insulin transport in endothelial cells appears to be vastly in caveolae [12], where CEACAM1 has recently been found to bind to caveolin-1 [13].

In endothelial cells, CEACAM1 maintains vascular integrity during physiologic neovascularization [14]. Upon its phosphorylation by VEGFR2 in response to VEGF-A, CEACAM1 regulates endothelial cell differentiation and barrier function [15]. Consistently, global *Cc1*^−/−^ mice displayed endothelial and vascular dysfunction [7] with endothelial barrier permeability related to the dissociation of VECadherin/β-catenin complexes at inter-endothelial junctions [13]. Aortae [16] and myocardial endothelial cells [13] of *Cc1*^−/−^ mice exhibited reduced glycocalyx thickness to confer more leakiness of the endothelial barrier. In contrast, mice with endothelial-specific CEACAM1 overexpression display ensheathment of the endothelial barrier by pericytes [17], lending support to the cell-autonomous role of endothelial CEACAM1 in maintaining vascular integrity.

Given the role of the endothelial barrier in regulating insulin transport to extrahepatic target tissues [9], we questioned whether endothelial CEACAM1 regulates peripheral insulin action independently of its regulation of insulin clearance in hepatocytes. To this end, we generated mice with endothelial cell-specific *Ceacam1* null deletion and characterized its metabolic phenotype using hyperinsulinemic-euglycemic clamp analysis in addition to determining the molecular and cellular basis of insulin action in peripheral insulin target tissues.

## 2. Materials and Methods

### 2.1. Generation of Null Mice

C57BL/6J *VECadherinCre+Cc1^fl/fl^* mice (*VECadCre+Cc1^fl/fl^* or VECad+*Cc1^fl/fl^*) were generated as described for liver-specific null mice [5] and genotyped as detailed in Supplementary Materials (Figure S1). Briefly, *Cc1*^loxp/loxp^ mice were crossed with C57BL/6J transgenic mice expressing Cre under the transcriptional control of the VECadherin promoter (*VECadherin Cre*) (Jackson Laboratories, Bar Harbor, ME). Littermate controls included: homozygous mice with wild-type *Ceacam1* allele with *VECadCre* (*VECadCre+Cc1^+/+^*) or without (*VECadCre−Cc1^+/+^*), and homozygotes with *Ceacam1*-floxed allele with (*VECadCre+Cc1^fl/fl^*) or without *VECadherin Cre* (*VECadCre–Cc1^fl/fl^*). Littermates were used to mitigate the confounding effects of introducing flox and *VECadherin Cre*.

Mice were housed in pathogen-free conditions and fed *ad libitum* a standard chow (Harlan Teklad 2016; Harlan, Haslett, MI, USA). The Animal Care and Utilization Committee of each participating institution approved all experiments.

### 2.2. Biochemical Parameters

To determine insulin clearance at a steady state, mice were fasted from 1700 h until 1100 h the next day, and their retro-orbital venous blood was drawn into heparinized micro-hematocrit capillary tubes (Fisherbrand, Waltham, MA, USA), as previously optimized [6]. Plasma was processed and analyzed for insulin (80-INSMSU-E01, Alpco, Salem, NH, USA), C-peptide (80-CPTMS-E01, Alpco), adiponectin (ab108785, Abcam, Cambridge, MA, USA), non-esterified fatty acids (NEFA-C colorimetric assay; Wako, Richmond, VA, USA), tumor necrosis factor-alpha and interleukin-6 (ELISA Kits, Abcam), and nitric oxide (NO) (Nitrate/Nitrite fluorometric assay Kit; Cayman Chemical, Ann Arbor, MI, USA). To determine plasma leptin levels, mice were fasted up to 4 h in the morning before blood was drawn (Linco Research, Billerica, MA, USA).

### 2.3. Glucose and Insulin Tolerance Tests

Awake 6 h-fasted mice were injected intraperitoneally with 1.5 g/kg BW dextrose solution or 0.75 units/kg BW human regular insulin (Novo Nordisk, Princeton, NJ, USA) before their tail blood glucose was measured at 0–180 min post-injection [18].

### 2.4. Hyperinsulinemic-Euglycemic Clamp Analysis

Awake overnight-fasted mice (*n* ≥ 8/genotype) were subjected to a 2 h-hyperinsulinemic-euglycemic clamp with primed and continuous infusion of human regular insulin (Humulin, Lilly, Indianapolis, IN, USA) at 2.5 mU/kg/min [18]. Glucose production/turnover was assessed by a continuous infusion of 0.05 μCi/min of [3-^3^H] glucose (PerkinElmer and Analytical Sciences, Hopkinton, MS, USA) followed by 0.1 μCi/min throughout the clamp.

### 2.5. Isolation of Primary Cells

Primary endothelial cells, hepatocytes, and bone marrow-derived macrophages were isolated from ketamine/xylazine-anesthetized 2 to 3-month-old mice [7,19]. Briefly, endothelial cells were isolated from the livers of 2-month-old mice by collagenase A (2 mg/mL) in CaCl_2_/MgCl_2_-supplemented Dulbecco’s PBS (Gibco Lab., Gaithersburg, MD, USA) and sorted by anti-mouse CD31 and immobilized on Dynabeads-Magnetic Particle Concentrator DynaMag^TM^-15 (Invitrogen, Carlsbad, CA, USA) [7]. Following resuspension in growth medium (DMEM (Gibco), 0.01% heparin, 10% bovine endothelial cell growth supplement—ECGS (Cell Applications Inc., Weathers Place San Diego, CA, USA), 20% FBS, and 1% penicillin-streptomycin), cells were plated on a 10 cm 0.1% gelatin-coated plates and incubated at 37 °C-5%CO_2_. Medium was changed every 2 days until cells reached ~80–90% confluency, trypsinized and incubated with rat anti-mouse CD102-coupled Dynabeads, immobilized, resuspended, and plated again in coated plates.

Hepatic stellate cells (HSCs) were isolated from mice of ≥8 months of age [20]. Briefly, livers were perfused with pronase (Sigma-Aldrich, St. Louis, MO, USA) solution followed by collagenase D (Roche Diagnostics, Indianapolis, IN, USA) and purified by density gradient-mediated separation using Nycodenz solution (Accurate Chemicals, Westbury, NY, USA). Cells were maintained at 37 °C-5%CO_2_ in DMEM-10% fetal bovine serum, 1% gentamycin, and 1% antibiotic/antimycotin (Gibco).

### 2.6. Insulin Treatment and Media Analysis

Primary liver endothelial cells (LEC) were seeded into 6-well plates at a density of 3 × 10^5^ cells/well in complete DMEM-F12 medium (Gibco) for 24 h. They were then serum-starved in phenol red-free medium (Invitrogen)-25 mM HEPES and 0.1% BSA for 2 h before treating with BSA-free insulin (100 nM in 25 mM HEPES) or vehicle at 37 °C for 5 min (for Western blot analysis of cell lysates) or for 20 min to analyze NO levels in 20 µL of media. Fluorescence was read using the Synergy H1 hybrid microplate reader (BioTek Instruments, Winooski, VT, USA) at 360 nm excitation and 430 nm emission wavelengths [21].

### 2.7. Western Blot Analysis

Protein lysates of tissues or LEC were subjected to Western blot analysis using 1:1000 of polyclonal antibodies from Cell Signaling (Danvers, MA, USA): anti-phospho-Akt (Ser473), Akt, phospho-p44/42 MAPK (Thr202/Tyr204), p44/42 MAPK, phospho-eNOS (Ser1177), eNOS, phospho-IRS1 (Ser612), IRS1, phospho-NF-κB p65 (Ser536), NF-κB, Shc, VECadherin, VEGFR2, VCAM1, ADAM10, and SHP2. The aforementioned SHP2. IRS1 and Shc antibodies were also used in co-immunoprecipitation experiments. Anti-phospho-insulin receptor-beta subunit (pIRβ) (phospho-Y1361) and IRβ from Abcam and anti-insulin receptor-alpha rabbit polyclonal antibody from Santa Cruz Biotechnology (Dallas, TX, USA) were also used. As previously shown [22,23], We also used a custom-made rabbit polyclonal antibody (Ab 3759) against mouse CEACAM1 extracellular domain and phospho-CEACAM1 (α-pCC1) mouse antibody (Bethyl Laboratories, Montgomery, TX, USA). For normalization, α-tubulin antibody (Cell Signaling, Danvers, MA, USA) or GAPDH antibody (Santa Cruz Biotechnology, Dallas, TX, USA) were used at 1:5000 dilution to immunoblot either the lower half of the same membrane or parallel gels. For signaling pathways, proteins were immunoblotted with antibodies against phosphorylated proteins and normalized by immunoblotting parallel gels with specific antibodies against these proteins. Following incubation with horseradish peroxidase (HRP)-conjugated sheep anti-mouse IgG or donkey anti-rabbit IgG antibody (GE Healthcare Life Sciences, Amersham, Sunnyvale, CA, USA), proteins were detected by enhanced chemiluminescence (Amersham). To prevent antibody heavy chain detection in co-immunoprecipitated complexes, membranes were immunoblotted with monoclonal mouse anti-rabbit HRP-conjugated IgG fraction (Jackson Immuno-Research Laboratories; #211-032-171).

### 2.8. Semi-Quantitative Real-Time PCR (qRT-PCR) Analysis of mRNA

Total RNA was isolated from primary cells, livers, and skeletal muscle with a NucleoSpin RNA Kit (740955.50, Macherey-Nagel, Bethlehem, PA, USA) and from visceral white adipose tissue (WAT) using TRIzol reagent (15596026, Ambion, Life Technologies, CA, USA). cDNA was synthesized using Superscript III (Bio-Rad, Hercules, CA, USA), and qRT-PCR was performed using Fast SYBR Green Master Mix by the ABI StepOnePlus Real-Time PCR System (Applied Biosystems, Beverly, MA, USA), as described [18]. All primers (Table S1) were used at a final concentration of 10 µM. mRNA was normalized relative to ribosomal 18S or 36B4 (for white adipose tissue). Values were expressed as mean ± SEM.

### 2.9. Gomori’s Trichrome Staining

Per [24], WAT (*n* = 5/genotype) was paraffin-blocked, and sections deparaffinized, hydrated, and the antigens unmasked in Boui’s fluid at 56 °C for 45 min. Nuclei were stained with Working Weigert’s Iron Hematoxylin before undergoing trichrome staining using the Thermo Scientific Richard-Allan Scientific Chromaview-advanced Testing (N. 87020).

### 2.10. Immunofluorescence of Visceral White Adipose Tissue

As previously done [24], WAT was formalin-fixed (Z-fix formalin, Anatech, Battle Creek, MI, USA), permeabilized in 1% Triton X-100 (Thermo Fisher Scientific, Walthman, MA, USA) in PBS for 10 min before staining with rat anti-mouse F4/80 monoclonal antibody (Thermo Fisher Scientific) followed by donkey anti-rat IgG conjugated to Alexa Fluor488 (Invitrogen) to detect macrophages. To visualize lipid, tissues were left rocking overnight at room temperature (RT), washed in PBS, incubated in secondary stain for 2 h at RT, washed and counterstained with 5 µM BODIPY 558/568 (Molecular Probes, Inc, Eugene, OR, USA) for 15 min at RT to visualize lipid. To mark CEACAM1, a custom-made affinity-purified rabbit anti-mouse polyclonal antibody (Bethyl Laboratories) was used at 1:100 dilution in PBS/1% BSA, followed by FITC-conjugated goat anti-rabbit antibody (Abcam). For staining of M2 macrophages, allophycocyanin (APC) anti-mouse CD301 (MGL1/MGL2) rat monoclonal antibody (BioLegend, San Diego, CA, USA) was used at a 1:100 followed by Texas Red-conjugated goat anti-rat IgG antibody (1:1000; Invitrogen). For M1 macrophages, anti-CD11c Armenian hamster anti-mouse monoclonal antibody (1:100; Abcam) and the secondary was Dylight 649 goat anti-Armenian hamster antibody (Invitrogen) at 1:1000 dilution. For imaging, samples were placed on a coverslip and visualized using a Leica TCS SP5 laser-scanning microscope (Leica Microsystems, Buffalo Grove, IL, USA) [24].

### 2.11. Statistical Analysis

Data were analyzed using one-way ANOVA analysis with post-hoc Tukey’s test for multiple comparisons, or two-tailed Student’s *t*-test, using GraphPad Prism6 software. Data were presented as mean ± SEM. *p* < 0.05 was considered statistically significant.

## 3. Results

### 3.1. Specific Deletion of Ceacam1 in Endothelial Cells of VECadCre+Cc1^fl/fl^ Mice

**Figure 1:**
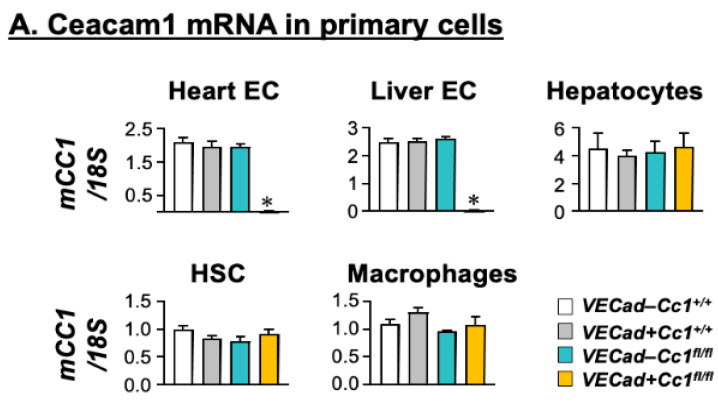

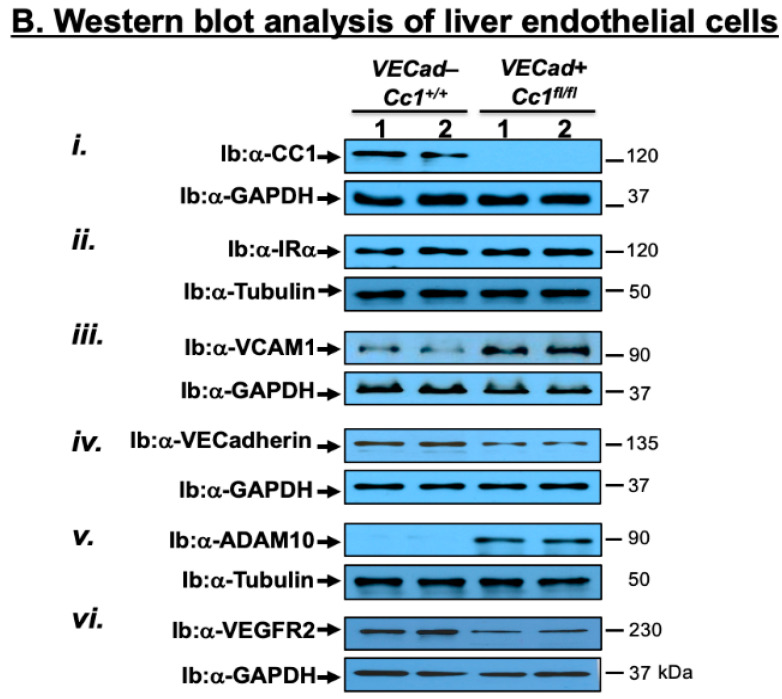
Assessing CEACAM1 expression in endothelial cells. (**A**) Primary cells were isolated from male mice at 2 months of age (*n* = 5/genotype), except for hepatic stellate cells that were derived from male mice at 8 months of age. Ceacam1 mRNA levels were analyzed by qRT-PCR in triplicate and normalized to 18S. Values are expressed as mean ± SEM. * *p* < 0.05 vs. all control groups; Negl, negligible. (**B**). Liver endothelial cells (LEC) combined from several wild-type (*VECad-Cc1^+/+^*) and null (*VECad+Cc1^fl/fl^*) mice were analyzed by immunoblotting (Ib) with specific antibodies (α-) to detect specific proteins and normalized against levels of loaded proteins by immunoblotting parallel gels or lower half-gels with α-GAPDH or α-Tubulin. The apparent molecular mass (kDa) is indicated at the right-hand side of each gel. Gels represent two separate/repeated experiments.

mRNA levels were determined by qRT-PCR analysis that demonstrated almost complete deletion of Ceacam1 from endothelial cells derived from the hearts and livers of *VECadCre+Cc1^fl/fl^* mice (Figure 1A). In contrast, Ceacam1 mRNA was intact in hepatocytes, hepatic stellate cells (HSC), and bone marrow macrophages (Figure 1A). Immunoblotting (Ib) cell lysates of liver endothelial cells (LEC) with CEACAM1 antibody (α-CC1) and normalizing against GAPDH to assess total protein loaded on the SDS-gel (Figure 1B) confirmed the loss of CEACAM1 in mutant LEC as compared to wild-type controls (Figure 1B(*i*)).

### 3.2. VECadCre+Cc1^fl/fl^ Mice Display Insulin Sensitivity

**Figure 2:**
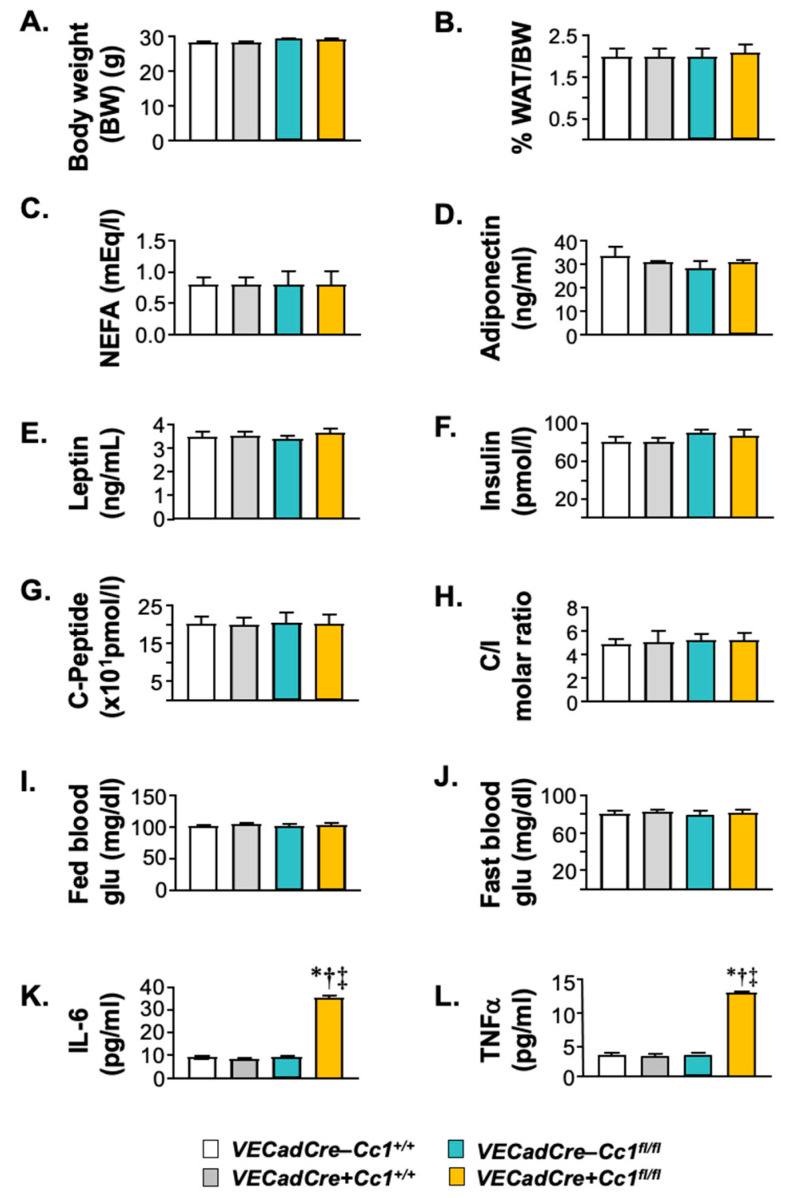
Biochemistry of plasma and tissues. Male mice (8 months of age, *n* ≥ 5/genotype) were fasted overnight from 5:00 p.m. until 11:00 a.m. the next morning before retro-orbital blood was drawn and tissues were excised. (**A**) body weight (BW). (**B**) % WAT/BW visceral adiposity measured as percentage of BW. Plasma levels of (**C**) non-esterified fatty acids (NEFA), (**D**) adiponectin, (**E**) leptin, (**F**) insulin, (**G**) C-peptide, (**K**) interleukin-6 (IL-6) and (**L**) tumor necrosis factor α(TNFα) were determined. (**H**) steady-state plasma C-peptide/insulin molar ratio (C/I) was calculated as measure of insulin clearance. Blood glucose (Glu) levels were determined at about 3–4 h of the beginning of the dark cycle (fed) (**I**) and fasting (**J**) states were also assessed. Values are expressed as mean ± SEM. * *p* < 0.05 vs. *VECadCre-Cc1^+/+^*, ^†^ *p* < 0.05 vs. *VECadCre+Cc1^+/+^*, ^‡^ *p* < 0.05 vs. *VECadCre-Cc1^fl/fl^*.

Compared to control littermates, *VECadCre+Cc1^fl/fl^* nulls exhibited normal body weight for up to 12 months of age (Figure 2A, data of 8-month-old mice are shown). This was associated with normal visceral adipose mass (Figure 2B). Consistently, plasma levels of NEFA, adiponectin, and leptin were also normal in nulls relative to control littermates (Figure 2C–E, respectively).

Null mice displayed normal steady-state plasma insulin (Figure 2F) and C-peptide (Figure 2G) levels. This was associated with normal insulin clearance (calculated as steady-state C-peptide/insulin molar ratio) (Figure 2H), consistent with the intact expression of CEACAM1 in hepatocytes, the major site of insulin clearance and home for the highest level of CEACAM1 expression [3].

**Figure 3:**
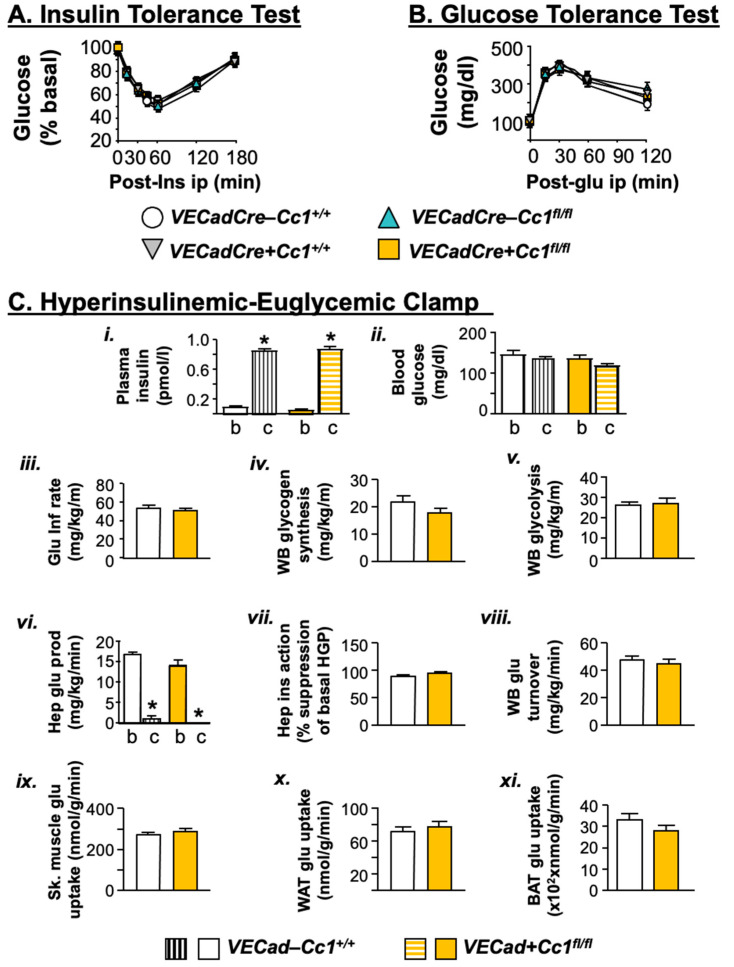
Metabolic phenotyping. Twelve-month-old male mice (*n* ≥ 7–8/genotype/age group) were injected intraperitoneally with insulin or glucose to assess insulin (**A**) and glucose tolerance (**B**). Values were expressed as mean ± SEM; *VECad−Cc1^+/+^* (wild-type controls), *VECad+Cc1^+/+^* (Cre controls), *VECad−Cc1^fl/fl^* (Flox controls) and *VECad+Cc1^fl/fl^* (null mice). (**C**) Hyperinsulinemic-euglycemic clamp analysis performed on 7-month-old awake overnight-fasted *VECad−Cc1^+/+^* and *VECad+Cc1^fl/fl^* nulls. *n* ≥ 8/genotype. Values are expressed as mean ± SEM. * *p* < 0.05 clamp (c) vs. basal (b). Glu Inf rate denotes glucose infusion rate, WB denotes whole body, Hep denotes hepatic, WAT denotes white adipose tissue, and BAT denotes brown adipose tissue.

In addition to normal plasma NEFA and adiponectin, null mice exhibited fed (and fasting) normoglycemia (Figure 2I,J), suggesting insulin sensitivity. Consistently, null mice remained tolerant to exogenous insulin and glucose until 12 months of age (Figure 3A,B, respectively). A 2 h-hyperinsulinemic/euglycemic (Figure 3C(*i*,*ii*)) clamp analysis was performed on 7-month-old mice. The glucose infusion rate required to maintain euglycemia (*iii*), whole-body glycogen synthesis (*iv*), and glycolysis (*v*) were normal in null mice relative to wild-type controls. The ability of insulin to suppress hepatic glucose production (*vi*,*vii*) and induce whole-body glucose turnover (Rd) (*viii*) and glucose uptake in the gastrocnemius muscle (*ix*) and in white and brown adipose tissue (*x*,*xi*) were all intact in *VECadCre+Cc1^fl/fl^* relative to *VECadCre-Cc1^+/+^* controls.

**Figure 4:**
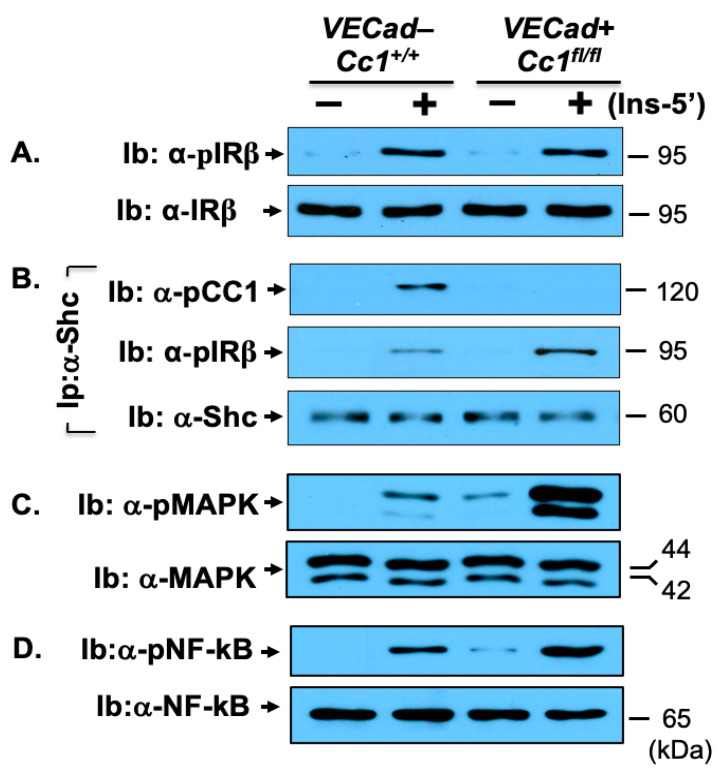
NF-κB insulin signaling pathway in liver endothelial cells. Primary liver endothelial cells (LEC) from 2-month-old male WT (*VECad−Cc1^+/+^*) and null (*VECad+Cc1^fl/fl^*) mice were treated with (Ins, 100 nM) or without insulin for 5 min before their lysates were subjected to immunblotting (Ib) with antibody against (**A**) phospho-IRβ (α-pIRβ), (**C**) phopsho-MAP kinase (α-pMAPK) and (**D**) phopho-NF-κB (α-pNF-κB) and in parallel gels, with their specific antibodies for normalization. (**B**) lysates were subjected to immunoprecipitation (Ip) with Shc antibody followed by immunoblotting (Ib) with antibodies against phospho-CEACAM1 (pCC1) and IRβ or Shc to assess the amount of pCC1 and pIRβ in the Shc immunopellet. Gels represent two separate experiments.

At the cellular level, Western blot analysis revealed that insulin induced IR beta (IRβ) phosphorylation to the same extent in mutant and wild-type LEC, as indicated by immunoblotting cell lysates with antibodies against phosphorylated IRβ (α-pIRβ) antibody normalized to IRβ (Figure 4A). Like IRβ, insulin receptor alpha (IRα) protein level was intact in LEC from null mice (Figure 1B(*ii*)).

### 3.3. Increased NF-κB-Mediated Systemic Inflammation in VECadCre+Cc1^fl/fl^ Mice

Null LEC (Figure 4D), as well as endothelial cells isolated from heart (not shown), exhibited a higher basal and insulin-stimulated NF-κB activation (phosphorylation) than wild-type LEC. This could result from the higher activation of MAP kinase both basally and in response to insulin (Figure 4C), which stemmed in turn, from lower Shc sequestration in the absence of CEACAM1 [25] and the reciprocal increase in its binding to IRβ as co-immunoprecipitation experiments revealed (Figure 4B).

NF-κB could drive the transcription and release of pro-inflammatory cytokines from LEC to contribute to elevated plasma IL-6 and TNFα levels in null mice starting at 8 months of age (Figure 2K,L, respectively). NF-κB could also induce the transcription of VCAM1 [26,27] to elevate its mRNA (by ~4-fold) (Table 1) and protein levels (Figure 1B(*iii*)), as well as the mRNA of ICAM1 and P-Selectin [28] (by ~3–5-fold) (Table 1); all being vascular mediators of leukocyte-endothelial adhesion.

The increase in VCAM1 protein level in *VECadherin+Cc1^fl/fl^* LEC is consistent with our previous findings in bovine aortic endothelial cells (BAEC) with siRNA-mediated downregulation of Ceacam1 [16]. Consistent with the inducing effect of NF-κB on the transcription of matrix metalloprotease (Mmp) 2 and 9 [29], mRNA levels were increased by ~2-fold in the heart and skeletal muscle of *VECadherin+Cc1^fl/fl^* relative to their three controls (Table 2). This could lower the mRNA of tight junctions and adherent molecules such as ZO-1/2, claudin 1/3, and occludin in null mice (Table 2) to contribute to endothelial barrier dysfunction in these tissues [30].

### 3.4. Compromised Endothelial Barrier and Lower Nitric Oxide Production in VECadCre+Cc1^fl/fl^ Mice

**Figure 5:**
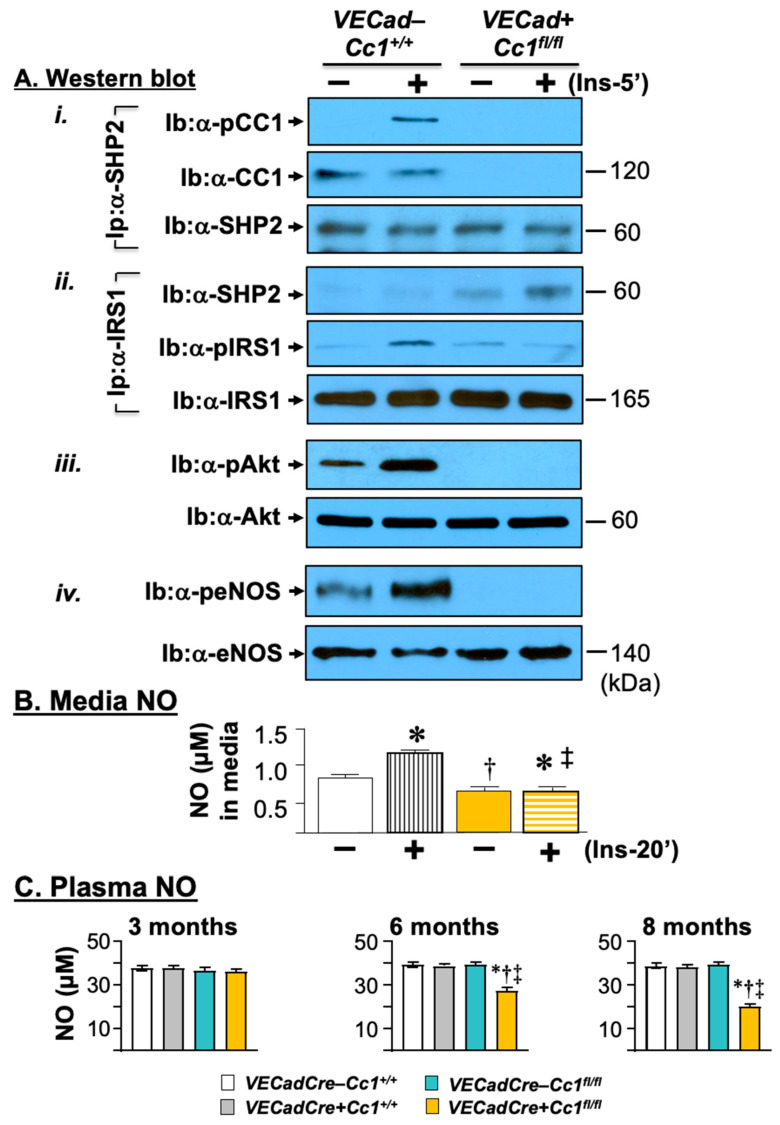
Insulin signaling leading to nitric oxide production in liver endothelial cells. (**A**) Primary liver endothelial cells (LEC) from 2-month-old WT (*VECad−Cc1^+/+^*) and null (*VECad+Cc1^fl/fl^*) mice were treated with (Ins, 100 nM) or without insulin for 5 min before proteins were lyzed and (*i*) subjected to immunoprecipitation (Ip) with SHP2 antibody followed by immunoblotting (Ib) with antibodies against phospho-CEACAM1 (pCC1) and CEACAM1 (CC1) or SHP2 to assess the amount of CC1 and pCC1 in the SHP2 immunopellet. (*ii*) Similar co-immunoprecipitation experiments were performed to evaluate the association between IRS1 with SHP2. (*iii*,*iv*) Lysates were also subjected to immunoblotting with α-phospho-antibodies to account for activation of Akt and eNOS, respectively, as in the legend in Figure 4. Gels represent two separate experiments. (**B**) levels of nitric oxide (NO) were determined in triplicate in the media of cells treated with or without insulin for 20 min. Values are expressed as mean ± SEM. * *p* < 0.05 vs. no insulin/same genotype; ^†^ *p* < 0.05 vs. *VECadCre−Cc1^+/+^* no insulin; ^‡^ *p* < 0.05 vs. *VECadCre−Cc1^+/+^* plus insulin.(**C**) Male mice (3–8 months of age, *n* ≥ 5/genotype) were fasted overnight before blood was drawn, and plasma was processed to assess NO levels. Values are expressed as mean ± SEM. * *p* < 0.05 vs. *VECadCre−Cc1^+/+^*, ^†^ *p* < 0.05 vs. *VECadCre+Cc1^+/+^*, ^‡^ *p* < 0.05 vs. *VECadCre−Cc1^fl/fl^*.

Consistent with CEACAM1 regulating endothelial barrier [15], the mRNA levels of β-catenin and other genes involved in adhesion and tight junctions (Vecadherin, occludin, ZO-1, claudin-1, and -5) critical to cell-to-cell contact, were all reduced by ~2-to-3-fold in null LEC (Table 1). VECadherin protein levels were lower in LEC (Figure 1B(*iv*)) from *VECadCre+Cc1^fl/fl^* mice. This could be partly caused by the rise in ADAM10 (Figure 1B(*v*)), a metalloprotease that cleaves VECadherin to promote vascular permeability and leukocyte trans-endothelial migration [31]. In addition, VEGFR2 protein levels were markedly reduced in null LEC (Figure 1B(*vi*)). qRT-PCR analysis showed ~three-to-five-fold lower levels of mRNA of Vegf-A and its receptor (Vegfr2) in addition to Vegfr1 in null LEC (Table 1). In contrast, mRNA levels of Vegf-C and Vegf-D that are mainly involved in lymphatic vessel formation were unaltered (Table 1). The differential downregulatory effect on Vegf-A transcription could be due, at least partly, to compromised Akt/eNOS signaling (Figure 5A(*iii,iv*)), leading to lower NO production and release in the media of LEC both basally and in response to insulin (Figure 5B), and to reduced β-catenin mRNA levels in LEC cells (Table 1) [32]. This would contribute to the significant drop in plasma NO levels in *VECadCre+Cc1^fl/fl^* mice beginning at 6 months of age (Figure 5C). Reduced Akt/eNOS phosphorylation could result from limited SHP2 phosphatase sequestration in the absence of CEACAM1 [16] and its reciprocal targeting to IRS-1, as co-immunoprecipitation analysis showed (Figure 5A(*i*,*ii*)).

### 3.5. Increased Fat Accumulation and Insulin Sensitivity in Adipocytes of VECadCre+Cc1^fl/fl^ Mice

**Figure 6:**
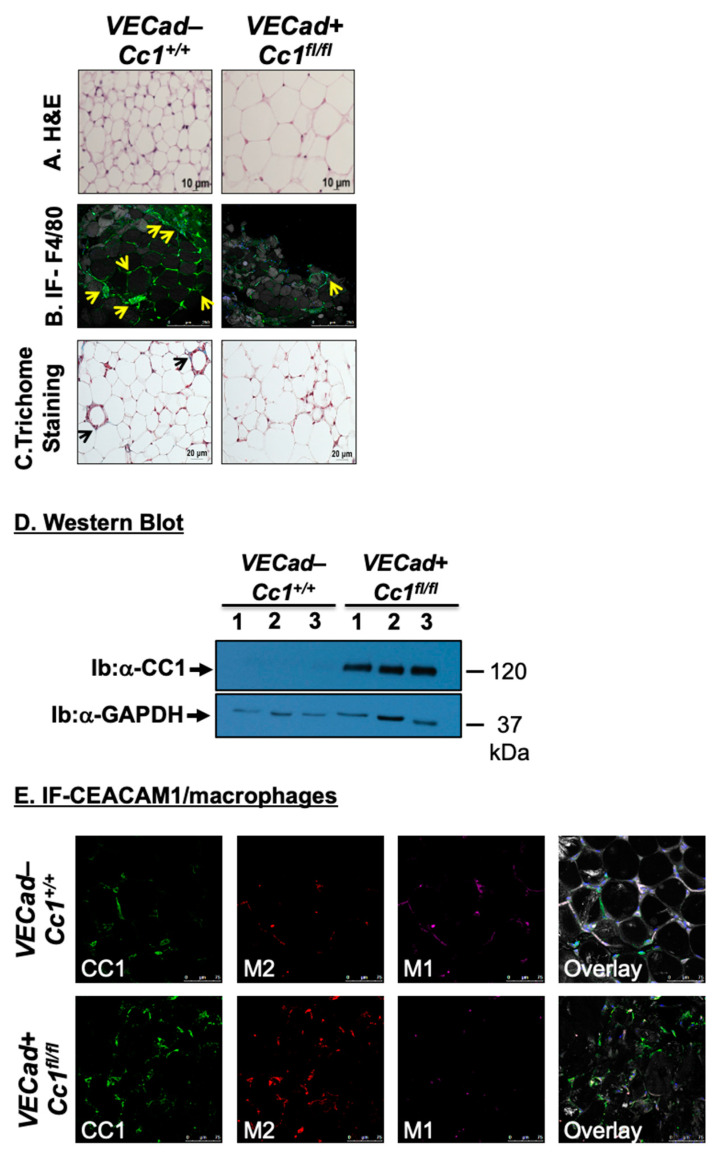
White adipose tissue histology and CEACAM1 expression. (**A**) White adipose tissue histology was assessed in H&E-stained sections (*n* > 4 mice/genotype). It indicates adipocytes’ expansion in KO versus WT. Representative images from three sections per mouse are shown. Scale bar: 10 µm. (**B**) Whole WAT was stained with BODIPY 558/568 to detect lipid (gray) and with anti-F4/80 to detect macrophages (green) and DAPI (blue) to detect nuclei. All images were captured using LSCM with a 20× objective and are 2D projections of a 3D image z-stack. Scale bar: 250 µm. Yellow arrows show multiple areas with crown-like structures (CLS) containing macrophages (green) surrounding adipocytes (gray). Small pieces of lipid are visible inside the macrophages of wild-type (left panels). In the right panel, a representative projection from KO mice is shown. A yellow arrow points to the single lipid-free CLS detected in the image (yellow arrow) surrounding one adipocyte from KO mice. Representative images from three sections per mouse are shown. (**C**) White adipose tissue from 5 mice/genotype was analyzed by trichome staining to detect collagen deposition. Representative images from three sections per mouse are shown. Scale bar: 20 µm. Black arrows point to trichome blue-stained collagen deposits in wild-type mice than in the EndoCC1 KO mice. (**D**) WAT lysates from 8-month-old mice were analyzed by immunoblotting the upper half of the membrane with α-CEACAM1 (CC1) antibody and normalizing the lower half with α-GPADH antibody to normalize against the total amount of proteins loaded. Gels represent two separate/repeated experiments. (**E**). Immunofluorescence analysis of white adipose tissue. Whole WAT tissue from WT and KO mice was stained with anti-CC1 (green) with APC anti-mouse CD301 (MGL1/MGL2) for M2 macrophages (red) and with anti-CD11c hamster anti-mouse antibody for M1 macrophages (magenta). The last panel is the overlay of all of the antibodies under consideration. All images were captured using LSCM with a 20× objective and are 2D projections of a 3D image z-stack. Scale bar: 75 µm. Representative images from three sections per mouse are shown.

Histological analysis of H&E-stained sections from WAT showed a significant expansion of adipocytes in 12-month-old *VECadCre+Cc1^fl/fl^* mice relative to their age-matched wild-type counterparts (Figure 6A). Immunofluorescence (IF) analysis with anti-F4/80, a macrophage marker, showed multiple areas with crown-like structures (CLS)-containing macrophages (green) surrounding adipocytes (gray) with small pieces of lipid inside the macrophages in wild-type mice (Figure 6B, yellow arrows). In contrast, *VECadCre+Cc1^fl/fl^* mice exhibited minimal CLS formation with no evidence of lipids inside the macrophages in these structures.

Given that TNFα can induce CEACAM1 transcription via activating NF-κB [33], we then examined CEACAM1 mRNA and protein levels in WAT. As expected from the low expression of CEACAM1 in WAT under physiological conditions [34], Western blot analysis did not detect CEACAM1 protein in wild-type WAT lysates (Figure 6D). In contrast, WAT from *VECadCre+Cc1^fl/fl^* mice exhibited a substantial increase in CEACAM1 protein content (Figure 6D). This appears to be due to a preferential ~2-fold increase in the mRNA of Ceacam1-4L, but not its alternative spliced variant, Ceacam1-4S [35], as expected from elevated levels of interferon response factor (Irf)-1 [36] and Irf-3 [37] in the WAT of null mice relative to their controls (Table 2). Consistently, IF analysis of CEACAM1 revealed a 2.5-fold increase in CEACAM1 expression (green) in *VECadCre+Cc1^fl/fl^* than wild-type WAT, and its parallel increase in its co-localization with the anti-inflammatory M2 macrophages (red), as the overlay revealed (Figure 6E). In contrast, wild-type WAT showed more of the pro-inflammatory M1 macrophages (magenta) than the null (Figure 6E). This is consistent with the anti-inflammatory function of CEACAM1-4L that is mediated by two immunoreceptor tyrosine-based inhibitory motifs in its cytoplasmic tail [38]. Accordingly, the mRNA levels of the anti-inflammatory IL-10 cytokine were elevated in null WAT (Table 2) without any change in the expression of pro-inflammatory cytokines such as IL-6, TNFα, IL-1β, and IFNγ, and in the T regulatory FoxP3 in null by comparison to wild-type WAT (Table 2). Consistent with normal TNFα levels, Smad7 expression was intact in null WAT, likely suggesting an unaltered TGFβ signaling pathway, which could, in turn, maintain normal mRNA levels of pro-fibrogenic genes, such as α-Sma, Col1α1, and Col6α3 in WAT of null mice (Table 2). Furthermore, Trichome staining revealed a modest decline in collagen deposition in WAT of *VECadCre+Cc1^fl/fl^* relative to wild-type mice (Figure 6C).

## 4. Discussion

In hepatocytes, CEACAM1 promotes insulin sensitivity by increasing the rate of receptor-mediated insulin uptake followed by degradation [4]. Accordingly, liver-specific and global *Cc1*^−/−^ null mice developed chronic hyperinsulinemia and insulin resistance emanating from impaired insulin clearance. *Cc1*^−/−^ also manifested endothelial and cardiovascular dysfunction and leukocyte-endothelial adherence [7]. Exclusive reconstitution of CEACAM1 in hepatocytes of *Cc1*^−/−^ mice reversed these metabolic [8] and cardiovascular abnormalities, including restoration of NO bioavailability and stemming endothelial-leukocyte adhesion [7]. This emphasized the significant contribution of hyperinsulinemia-driven insulin resistance in endothelial and cardiovascular dysfunction.

In contrast to liver-specific *Ceacam1* deletion or inactivation, the current studies showed that endothelial loss of Ceacam1 did not alter insulin metabolism or sensitivity, as buttressed by normoinsulinemia and normoglycemia (both fed and fasting) in *VECadherin+Cc1^fl/fl^* mice. Consistently, insulin receptor expression and phosphorylation in isolated liver endothelial cells were not impacted by the loss of endothelial CEACAM1. This is in contrast to the remarkable hyperinsulinemia-driven downregulation of insulin receptors in the hepatocytes of liver-specific inactive CEACAM1 mutants, as analyzed by Scatchard plot [6]. Normal insulin metabolism in *VECadherin+Cc1^fl/fl^* mice is consistent with: (1) the rapid and passive transport of secreted insulin from the portal vein to hepatocytes through fenestrae in the liver endothelium to be targeted to degradation, (2) intact CEACAM1 in hepatocytes, main sites of insulin extraction and (3) insulin is not significantly degraded in endothelial cells [11].

Ablating insulin receptors in endothelial cells adversely affected PI3-kinase-dependent insulin signaling pathways without altering glucose homeostasis under normal feeding conditions [39]. Whereas deleting endothelial *Ceacam1* did not affect insulin-stimulated insulin receptor phosphorylation in isolated liver endothelial cells, it deactivated the IRS-1/Akt/eNOS pathway to reduce eNOS-mediated NO production, a hallmark of endothelial dysfunction, as was manifested in global *Cc1*^−/−^ nulls by reduced endothelium-dependent relaxation in aortae [16] and microvasculature [7]. As previously shown in BAEC with siRNA-mediated downregulation of *Ceacam1* [16] and in the heart and aortae of *Cc1*^−/−^ mice [7], lowered eNOS activity was mediated by reduced sequestration of SHP2 phosphatase and its reciprocal increased binding to IRS-1. Recent studies in isolated myocardial cells from *Cc1*^−/−^ mice showed that compromised eNOS activity was also mediated by its depalmitoylation and increased caveolae-mediated redistribution to the perinuclear region [13]. Lower NO production would be expected to cause vasoconstriction, as in global *Cc1*^−/−^ mice [7], which would, in turn, limit blood flow and insulin delivery to skeletal muscle where insulin transport into the interstitial space is tightly regulated by the endothelial lining of its vasculature [40]. However, hyperinsulinemic-euglycemic clamp analysis showed normal insulin-stimulated glucose uptake in skeletal muscle (and adipose tissue) of *VECadherin+Cc1^fl/fl^* mice. Thus, it is likely that counterregulatory mechanisms developed to limit the potential adverse effect of vasoconstriction on insulin delivery to their extrahepatic target cells. Insulin is transported to myocytes either by a transcytosis mechanism inside the endothelial cell, or via a paracellular route through pores between adjacent endothelial cells [9]. Whether the loss of endothelial CEACAM1 impacted transcellular insulin access to the interstitial space, as expected from the loss of NO production [41] in endothelial cells isolated from *VECadherin+Cc1^fl/fl^* mice, remains to be determined. However, like myocardial endothelial cells of *Cc1*^−/−^ mice that exhibited destabilization of VECadherin/β-catenin complexes at the adherent junctions [13], *VECadherin+Cc1^fl/fl^* endothelial cells displayed a loss of VECadherin and β-catenin relative to wild-type mice. This is consistent with increased endothelial barrier dysfunction and vascular leakiness, which is expected to be intensified by the simultaneous rise of the inflammatory microenvironment caused by NF-κB-dependent production of TNFα in *VECadherin+Cc1^fl/fl^* mice [42]. Moreover, the loss of endothelial CEACAM1 yielded an increase in Shc binding to IRβ and activation of its downstream NF-κB pathway that would in turn, drive the transcription and release of MMP2 and 9 [29] in the heart and skeletal muscle of *VECadherin+Cc1^fl/fl^* relative to their three controls. This could lower the expression of tight junctions and adherent molecules (i.e., ZO-1/2, claudin 1/3 and occludin) in null mice to contribute to endothelial barrier dysfunction in extrahepatic tissues [30]. Together, the data suggest that like endothelial cells from global *Cc1*^−/−^ mice [13], *VECadherin+Cc1^fl/fl^* developed vascular permeability that would facilitate paracellular transport of insulin (~6 kDa) in extrahepatic peripheral tissues to support their normal glucose homeostasis.

*VECadherin+Cc1^fl/fl^* mice displayed an expansion of adipocytes to accommodate the storage of extra fat that is likely transported through the more permeable endothelial cell barrier [43,44]. However, these mice did not display a significant increase in visceral obesity, in association with normal plasma NEFA, adiponectin, and leptin levels. Like skeletal muscle, insulin-stimulated glucose uptake in white and brown adipose tissue was intact in *VECadherin+Cc1^fl/fl^* mice, as opposed to global *Cc1*^−/−^ mice. This is consistent with the rise in plasma TNFα and IL-6 emanating mostly from NF-κB activation in endothelial cells without involving adipokines released from adipose tissue macrophages that are typically associated with systemic insulin resistance and visceral obesity [45]. Normal plasma NEFA may have played an important part in insulin sensitivity, as supported by studies showing that treatment with acipimox, a lipolysis inhibitor, restored insulin’s suppression of endogenous glucose production without altering adipokines (IL-6 and TNFα) or adiponectin levels in subjects with a strong family history of type 2 diabetes [46]. The rise in plasma TNFα may have served to preferentially induce the expression of the Ceacam1-4L variant through activating NF-κB in the anti-inflammatory M2 macrophages of *VECadherin+Cc1^fl/fl^* to enhance innate immunity and limit systemic inflammation that would otherwise drive insulin resistance. The resultant low-level inflammation in the adipocytic microenvironment of *VECadherin+Cc1^fl/fl^* would limit fibrosis to bestow plasticity and enhance the expansion of adipocytes to store excess fat [47]. While we have observed that overexpression of CEACAM1 in hepatocytes limits fibrosis in adipose tissue [24], the current studies provide a first in vivo demonstration of the anti-fibrogenic role of CEACAM1-4L in M2 macrophages.

## 5. Conclusions

In summary, deleting *Ceacam1* in endothelial cells did not adversely affect systemic insulin sensitivity despite modulating post-receptor signaling to reduce NO production in addition to stimulating an NF-κB-dependent pro-inflammatory microenvironment in the endothelium. Sustained insulin sensitivity is in part due to intact insulin metabolism and increased fat accumulation in adipocytes. The studies provide an in vivo demonstration of a distinct endothelial cell CEACAM1-dependent pathway playing a role in regulating insulin sensitivity in extrahepatic cells independent of its role in insulin clearance in hepatocytes.

## Figures and Tables

**Table 1:** mRNA levels of genes in primary liver endothelial cells.

	*VECadCre−Cc1^+/+^*	*VECadCre+Cc1^fl/fl^*
**Cell adhesion**		
*Vcam1*	1.51 ± 0.11	6.24 ± 0.55 *
*Icam1*	1.01 ± 0.19	5.34 ± 0.15 *
*P-Selectin*	1.34 ± 0.12	3.72 ± 0.57 *
**Adherence/Tight junctions**		
*Vecadherin*	5.12 ± 1.28	2.15 ± 0.43 *
*β-Catenin*	7.23 ± 1.42	3.43 ± 0.87 *
*Occludin*	6.75 ± 1.22	3.21 ± 0.51 *
*ZO-1*	3.75 ± 0.56	1.04 ± 0.19 *
*Claudin1*	4.68 ± 1.13	1.52 ± 0.76 *
*Claudin5*	4.89 ± 1.31	1.67 ± 0.64 *
**Vascular permeability**		
*Vegf-A*	8.37 ± 1.68	2.45 ± 0.78 *
*Vegf-C*	1.56 ± 0.07	1.41 ± 0.09
*Vegf-D*	0.98 ± 0.12	0.95 ± 0.11
*Vegfr1*	2.01 ± 0.04	0.97 ± 0.03 *
*Vegfr2*	1.75 ± 0.08	0.37 ± 0.06 *

mRNA levels were assessed in liver endothelial cells from 2-month-old male mice (*n* = 5/genotype) by qRT-PCR in triplicate using gene-specific primers and normalized to 18S. Values are expressed as mean ± SEM. * *p* < 0.05 vs. *VECadCre−Cc1^+/+^*.

**Table 2:** qRT-PCR analysis of genes in tissues from 8-month-old mice.

	*VECadCre−Cc1^+/+^*	*VECadCre+Cc1^+/+^*	*VECadCre−Cc1^fl/fl^*	*VECadCre+Cc1^fl/fl^*
**Heart**				
*Mmp2*	1.99 ± 0.20	1.87 ± 0.15	1.80 ± 0.15	3.79 ± 0.20 *
*Mmp9*	1.11 ± 0.10	1.10 ± 0.11	1.15 ± 0.12	2.58 ± 0.15 *
*ZO-1*	2.86 ± 0.26	2.82 ± 0.25	3.79 ± 0.30	0.81 ± 0.20 *
*ZO-2*	2.99 ± 0.21	3.00 ± 0.18	3.05 ± 0.15	1.28 ± 0.15 *
*Claudin1*	1.47 ± 0.12	1.50 ± 0.16	1.40 ± 0.10	0.72 ± 0.09 *
*Claudin3*	2.58 ± 0.21	2.61 ± 0.25	2.65 ± 0.20	1.05 ± 0.15 *
*Occludin*	2.33 ± 0.20	2.53 ± 0.20	2.40 ± 0.25	1.06 ± 0.20 *
**Skeletal Muscle**				
*MMP2*	1.35 ± 0.16	1.31 ± 0.12	1.32 ± 0.12	3.43 ± 0.17 *
*MMP9*	0.81 ± 0.12	0.75 ± 0.10	0.84 ± 0.12	2.25 ± 0.10 *
*ZO-1*	3.57 ± 0.30	3.45 ± 0.27	3.38 ± 0.35	0.72 ± 0.30 *
*ZO-2*	1.25 ± 0.13	1.23 ± 0.18	1.84 ± 0.15	0.57 ± 0.12 *
*Claudin1*	2.18 ± 0.20	2.22 ± 0.15	2.15 ± 0.24	0.87 ± 0.20 *
*Claudin3*	0.72 ± 0.23	0.84 ± 0.20	0.75 ± 0.18	0.27 ± 0.13 *
*Occludin*	1.76 ± 0.18	1.79 ± 0.20	1.70 ± 0.15	0.60 ± 0.15 *
**WAT**				
*Ceacam1*	1.78 ± 0.15	1.79 ± 0.11	1.82 ± 0.13	3.00 ± 0.25 *
*Ceacam1-4L*	1.02 ± 0.15	0.97 ± 0.17	1.03 ± 0.15	2.32 ± 0.10 *
*Ceacam1-4S*	0.87 ± 0.09	0.77 ± 0.10	0.80 ± 0.10	0.82 ± 0.12
*Irf-1*	2.02 ± 0.12	2.19 ± 0.19	2.09 ± 0.10	3.92 ± 0.20 *
*Irf-3*	1.29 ± 0.15	1.27 ± 0.17	1.24 ± 0.09	2.79 ± 0.07 *
*Il-10*	1.02 ± 0.17	1.06 ± 0.13	1.05 ± 0.14	2.97 ± 0.09 *
*Foxp3*	1.43 ± 0.15	1.47 ± 0.16	1.40 ± 0.15	1.44 ± 0.20
*Tnfα*	0.48 ± 0.07	0.55 ± 0.09	0.51 ± 0.06	0.49 ± 0.10
*Il-6*	2.01 ± 0.18	1.98 ± 0.23	2.05 ± 0.21	2.10 ± 0.24
*Il-1β*	1.75 ± 0.34	1.78 ± 0.30	1.84 ± 0.27	1.70 ± 0.19
*Ifn*γ	1.91 ± 0.02	1.94 ± 0.08	1.98 ± 0.02	1.95 ± 0.08
*Smad7*	1.00 ± 0.25	1.03 ± 0.31	1.10 ± 0.28	1.04 ± 0.29
*α-Sma*	0.63 ± 0.12	0.65 ± 0.10	0.59 ± 0.15	0.60 ± 0.13
*Col1α1*	1.18 ± 0.05	1.17 ± 0.08	1.21 ± 0.11	1.21 ± 0.10
*Col6α3*	1.71 ± 0.21	1.77 ± 0.23	1.79 ± 0.19	1.75 ± 0.22

mRNA levels were analyzed in the hearts, skeletal muscle, and WAT of 8-month-old male mice (*n* = 5/genotype) by qRT-PCR in triplicate using gene-specific oligos and normalized to 18S. Values are expressed as mean ± SEM. * *p* < 0.05 vs. all three controls.

## Data Availability

The data presented in this study can be obtained upon a reasonable request to the corresponding author (S.M.N.).

## Supplementary Materials

The following supporting information is available online at https://www.mdpi.com/article/10.3390/cells10082093/s1, Figure S1: Mice genotyping, Table S1: Real-time PCR primer sequences from mouse genes.
