# Supplementary material for: Insulin Sensitivity Is Retained in Mice with Endothelial Loss of Carcinoembryonic Antigen Cell Adhesion Molecule 1
Source: Cells. 2021 Aug 14;10(8):2093. doi: 10.3390/cells10082093 (PMC8394790; doi:10.3390/cells10082093)
Supplement: Supplementary file 1 [file cells-10-02093-s001.zip › cells-1335662-supplementary.pdf]

Supplementary Material

Insulin Sensitivity is Retained in Mice with Endothelial Loss of Carcinoembryonic Antigen Cell Adhesion Molecule 1

Harrison T. Muturi[2,*], Saja S. Khuder[1,*], Hilda E. Ghadieh[2,*], Emily L. Esakov[1,3], Hye Lim Noh[4], Hee Joon Kang[4,6], Marcia F. McInerney[1,3], Jason K. Kim[4,5], Abraham D. Lee[7]; and Sonia M. Najjar[1,2,8,†]

[1] Center for Diabetes and Endocrine Research, College of Medicine and Life Sciences, University of Toledo, Toledo, OH, USA
[2] Department of Biomedical Sciences, Heritage College of Osteopathic Medicine, Ohio University, Athens, OH, USA
[3] Department of Medicinal and Biological Chemistry, College of Pharmacy and Pharmaceutical Sciences, Toledo, OH, USA
[4] Program in Molecular Medicine and [5] Division of Endocrinology, Metabolism and Diabetes, University of Massachusetts Medical School, Worcester, MA, USA.
[6] Department of Breast-Endocrine Surgery, Dongtan Sacred Heart Hospital, Hallym University College of Medicine, Seoul, South Korea
[7] Department of Rehabilitation Sciences, Judith Herb College of Education, Human Science and Human Service, The University of Toledo, Toledo, OH, USA
[8] Diabetes Institute, Heritage College of Osteopathic Medicine, Ohio University, Athens, OH, USA

* Authors of equal contributions

[†]Address correspondence to:
Prof. SONIA M. NAJJAR, Ph.D.
Prof and OHF John J. Kopchick Ph.D., Endowed Eminent Research Chair
Heritage College of Osteopathic Medicine.
Irvine Hall, 1 Ohio University
Athens, OH 45701-2979
Tel: 740-593-2376
Fax: 740-593-2778
Email: najjar@ohio.edu

METHODS AND MATERIALS

Genotyping of mice

C57BL/6J.*VECadherinCre+Cc1$^{fl/fl}$* (*VECadCre+Cc1$^{fl/fl}$*) mice were generated as described for Tcell-specific null mice (1). Briefly, the targeting construct inserted a loxP-neo cassette in intron 6 and a loxP fragment in intron 9, deleting a sequence that encodes the cytoplasmic domain (2). *Cc1*$^{loxp/loxp}$ mice were crossed with transgenic mice expressing Cre under the transcriptional control of the *VECadherin* promoter (*VECadCre*) on C57BL/6J background (Jackson Laboratories, Bar Harbor, ME). Heterozygous mice were backcrossed >6x with C57BL/6J mice. Using *Ceacam1 (Cc1)*-specific primers, offsprings were genotyped by PCR analysis of ear DNA (Figure. S1A) to identify homozygous mice with wild-type *Cc1* allele with *VECadCre* (*VECadCre+Cc1$^{+/+}$*) or without (*VECadCre–Cc1$^{+/+}$*), and with *Cc1*-floxed allele with *VECadCre* (*VECadCre+Cc1$^{fl/fl}$*) or without (*VECadCre–Cc1$^{fl/fl}$*). All homozygous lines were derived from the same breeding to mitigate potential confounding effects of floxing and introducing *VECadherin*.

The Flox gene was detected by a PCR reaction combining FloxA forward primer (FP) and FloxB and FloxC reverse primers (RP) (Figure. S1A). The FloxA/FloxB primer pair detected the 382bp wild-type allele (*Cc1$^{+/+}$*) and the FloxA/FloxC pair detected the 488bp null when the primer sets of the Cre reaction were combined: VECadherinCre FP, VECadherin Cre RP, and VECadherin gene primer. *VECadCre+* allele was identified by the detection of the 300bp *VECadherin* promoter and the 550bp *VECadherin* gene. *VECadCre–* allele was identified by detecting the 550bp *VECadherin* gene only. The same approach was used for bone marrow macrophages of homozygous mice (Fig. S1A). The PCR amplification yielded a 300bp product in gDNA isolated from the ear, but not macrophages of *VECadCre+Cc1$^{+/+}$* and *VECadCre+Cc1$^{fl/fl}$* mice when a primer set specific for *VECadCre* promoter was used. A 550bp DNA segment from *VECadherin* gene was amplified in the macrophages and ear lysates of all mice. As expected, a 488bp sequence from the Flox gene was only amplified in mice positive for *Cc1$^{flox/flox}$*, while a 382bp product was only detected in mice positive for wild-type allele.

PCR analysis of Ceacam1 alternative spliced isoforms in liver endothelial cells

Ceacam1 gene undergoes alternative splicing to yield 2 variants differing by the presence or absence of Exon 7 (53bp) that encodes a long (*Cc1-L*) and a short (*Cc1-S*) isoform (2, 3). To ascertain deletion of both isoforms in *VECadCre+Cc1$^{fl/fl}$* mice, endothelial cells were derived from liver (or heart) (Figure. S1B) and their cDNA was synthesized by iScript cDNA Synthesis Kit (Bio-Rad, Hercules, CA). A forward primer recognizing both variants (FP46) and two reverse primers, specific for each of the two spliced isoforms,

were used (4). The reverse primer for *Cc1-L* (BP43) recognized the alternatively spliced exon 7 and the reverse primer for *Cc1-S* isoform (BP44) was designed to anneal to the sequence across the splice junction between exon 6 and exon 8. PCR was initiated by denaturing at 94°C for 60s, followed by 30 cycles (94°C for 45s, 64°C for 45s, 72°C for 60s), and a final extension at 72°C for 10min. PCR products were analyzed by 2.7% agarose gels in sodium-borate buffer and visualized by ethidium bromide staining. The 658bp PCR product represents *Cc1-L* and the 626bp band designates *Cc1-S*. Nucleotide sequences of primers were listed in the table in Figure. S2B.

Table S1: Real-time PCR primer sequences from mouse genes

Primer	Forward Sequence (5'-3')	Reverse Sequence (5'-3')
β-Catenin	TCCCTGAGACGCTAGATGAGG	CGTTTAGCAGTTTTGTCAGCTC
Cc1 (Total)	AATCTGCCCCTGGCGCTTGGAGCC	AAATCGCACAGTCGCCTGAGTACG
Cc1-4L	GCGAGATCTCACAGAGCACA	GCTGGGAATTGAAGTTCAGG
Cc1-4S	CTGGCATCGTGATTGGAGTT	CAGAAGGAGCCAGATCCG
Claudin-1	TGAGCCTCAGAAAAGAGCC	GCCACTAATATCGCCAGACC
Claudin-3	CAGTGTACCAACTGCGTACAAGAC	ACCGGTACTAAGGTGAGCAGAG
Claudin-5	ATGGCGATTACGACAAGAAG	ACTGAGCAAATTCTTGCCC
Col1α1	TAGGCCATTGTGTATGCAGC	ACATGTTCAGCTTTGTGGACC
Col6α3	GTCAGCTGAGTCTTGTGCTGT	ACCTAGAGAACGTTACCTCACT
Foxp3	CCCAGGAAAGACAGCAACCTT	TTTCACAACCAGGCCACTTG
Gapdh	CCAGGTTGTCTCCTGCGACT	ATACCAGGAAATGAGCTTGACAAAGT
Icam1	CAATTTCTCATGCCGCACAG	AGCTGGAAGATCGAAAGTCCG
Ifnγ	ATGAACGCTACACACTGCATC	CCATCCTTTTGCCAGTTCCTC
Il-1β	CCCTGCAGCTGGAGAGTGTGG	TATTCTGTCCATTGAGGTGGAG
Il-6	CTTGGGACTGCCGCTGGTGA	TGCAAGTGCATCATCGTTGT
Il-10	CACAAAGCAGCCTTGCAGAA	AGAGCAGGCAGCATAGCAGTG
Irf-1	AACTTTGGCATTGTGGAAGG	ACACATTGGGGGTAGGAACA
Irf-3	ACTGCGTCTAGGCTGGTGGTTATT	TCTGGACCTGTCTTGTCCTTGCTT
Irf-8	CGTGGAAGACGAGGTTACGCTG	GCTGAATGGTGTGTGTCATAGGC
Mmp2	CAAGTTCCCCGGCGATGTC	TTCTGGTCAAGGTCACCTGTC

Mmp9	CTGGACAGCCAGACACTAAAG	CTCGCGGCAAGTCTTCAGAG
Occludin	CTTCTGCTTCATCGCTTCC	CTTGCCCTTTCCTGCTTTC
P-Selectin	TGCTGACTCCGGGCAGTGGA	GGTTGGCGGAGGTGCTGACC
α-Sma	CGTTGGCTATTCCTTCGTTAC	TGCCAGGAGACTCCATCC
Smad7	GTTGCTGTGAATCTTACGGG	ATCTGGACAGCCTGCA
Tnfα	CCACCACGCTCTTCTGTCTAC	AGGGTCTGGGCCATAGAACT
Vcam1	ATTTTCTGGGGCAGGAAGTT	ACGTGAGAACAACCGAATCC
VE-Cadherin	CACTGCTTTGGGAGCCTTC	GGGGCAGCGATTCATTTTTCT
Vegf-A	GCACATAGAGAGAATGAGCTTC	CTCCGCTCTGAACAAGGCT
Vegf-C	GTGAGGTGTGTATAGATGTGGG	GTCTTGCTGAGGTAACCTGTG
Vegf-D	TCACGCTCAGCATCCCATC	ACTTCTACGCATGTCTCTCTAGG
Vegfr1	AAGAGAGTCTGGCCTGCTTG	CTGCTCGGGTGTCTGCTT
Vegfr2	TTTGGCAAATACACCCCTTGAGA	GCAGAAGATACTGTCACCACC
ZO-1	ACAAACAGCCCTACCAACC	CCATCCTCATCTTCATCTTCTTC
ZO-2	GTTTTCTTCGTCCTAGTCCC	CATCCATCCCTTCCATCTTTC
18S	TTCGAACGTCTGCCCTATCAA	ATGGTAGGCACGGCGACT
36B4	GCAGACAACGTGGGCTCCAAGCAGAT	GGTCCTCCTTGGTGAACACGAAGCCC

A. Genotyping of mice

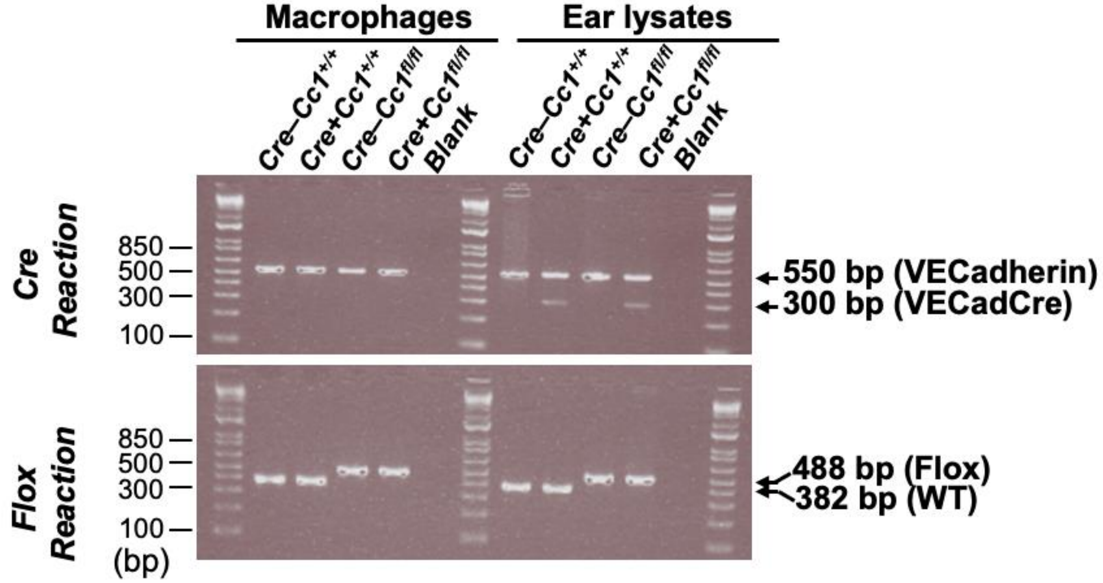

Gene	ID	Primer (5'-3')
Flox	FloxA FP	ACACAAGGAGGCCTCTCAGATGGCG
	FloxB RP	GACTTTGGCTTCCTGACTGGAGGA
	FloxC RP	GCGCCTCCCCTACCCGGTAGAATT
Cre	VECadCre FP	GCAGGCAGCTCACAAAGGAACAAT
	VECadCre RP	ATCACTCGTTGCATCGACCGGTAA
	VECadherin Gene	TGTCCTTGCTGAGTGACAGTGGAA

B. cDNA analysis in liver endothelial cells

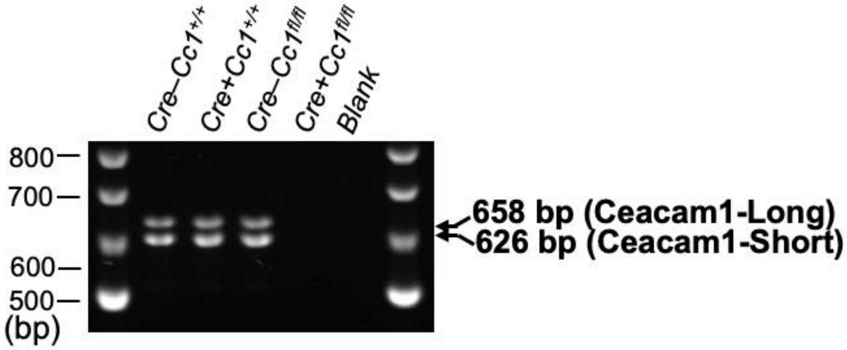

Gene	ID	Primer (5'-3')
Ceacam1	FP46	GCCATGCAGCCTCTAACCCACC
Ceacam1-4L	RP43	CTGGAGGTTGAGGGTTTGTGCTC
Ceacam1-4S	RP44	TCAGAAGGAGCCAGACCCGCC

Figure S1. Mice Genotyping. (**A**) To determine specificity of *Ceacam1* deletion in endothelial cells, but not macrophages, PCR amplification was performed on DNA from ear and macrophages. This yielded a 300bp product in ear DNA, but not macrophages of *VECadCre+Cc1*$^{+/+}$ and *VECadCre+Cc1*$^{fl/fl}$ mice. In contrast, a 550bp DNA segment from *VECadherin* gene was amplified in the macrophages and ear lysates of all mice. The Flox gene was detected by a PCR reaction combining FloxA forward primer (FP) and Flox B and C reverse primers (RP): The FloxA/FloxB primer pair detected the 382bp wild-type *Ceacam1* allele (*Cc1*$^{+/+}$) and the FloxA/FloxC pair detected the 488bp null allele (*Cc1*$^{fl/fl}$). As expected, a 488bp sequence from the Flox gene was only amplified in mice positive for *Cc1*$^{flox/flox}$, while a 382bp product was only detected in mice positive for wild type allele. Nucleotide sequences are listed in the table at the bottom of the illustration. **(B)** To examine whether both of *Ceacam1* gene spliced variants (*Cc1-L* and *Cc1-S*) differing by alternative splicing of Exon 7 were deleted, cDNA from liver primary endothelial cells was synthesized, and a PCR reaction using a forward primer recognizing both variants (FP46) paired up with two variant-specific reverse primers. RP43 from exon 7 was used to detect *Cc1-L* and RP44 annealing across the splice junction between exon 6 and exon 8 was used to detect *Cc1-S*. PCR analysis on 2.7% agarose gels showed deletion of both isoforms in the endothelial cells of *VECadCre+Cc1*$^{fl/fl}$ mice relative to the three controls.

References

1. Nagaishi T, Pao L, Lin SH, Iijima H, Kaser A, Qiao SW, Chen Z, et al. SHP1 phosphatase-dependent T cell inhibition by CEACAM1 adhesion molecule isoforms. Immunity 2006;25:769-781.

2. Najjar SM, Accili D, Philippe N, Jernberg J, Margolis R, Taylor SI. pp120/ecto-ATPase, an endogenous substrate of the insulin receptor tyrosine kinase, is expressed as two variably spliced isoforms. J. Biol. Chem. 1993;268:1201-1206.

3. Nédellec P, Turbide C, Beauchemin N. Characterization and transcriptional activity of the mouse biliary glycoprotein 1 gene, a carcinoembryonic antigen-related gene. Eur. J. Biochem. 1995;231:104-114.

4. Greicius G, Severinson E, Beauchemin N, Obrink B, Singer BB. CEACAM1 is a potent regulator of B cell receptor complex-induced activation. J. Leukoc. Biol. 2003;74:126-134.
